# Calculation of Bandwidth of Multimode Step-Index Polymer Photonic Crystal Fibers

**DOI:** 10.3390/polym13234218

**Published:** 2021-12-01

**Authors:** Branko Drljača, Svetislav Savović, Milan S. Kovačević, Ana Simović, Ljubica Kuzmanović, Alexandar Djordjevich, Rui Min

**Affiliations:** 1 Faculty of Sciences and Mathematics, University of Priština in Kosovska Mitrovica, L. Ribara 29, 38220 Kosovska Mitrovica, Serbia; branko.drljaca@pr.ac.rs; 2 Faculty of Science, University of Kragujevac, R. Domanovića 12, 34000 Kragujevac, Serbia; savovic@kg.ac.rs (S.S.); kovac@kg.ac.rs (M.S.K.); asimovic@kg.ac.rs (A.S.); ljubica.kuzmanovic@pmf.kg.ac.rs (L.K.); 3 Department of Mechanical Engineering, City University of Hong Kong, 83 Tat Chee Avenue, Hong Kong, China; mealex@cityu.edu.hk; 4 Center for Cognition and Neuroergonomics, State Key Laboratory of Cognitive Neuroscience and Learning, Beijing Normal University at Zhuhai, Zhuhai 519087, China

**Keywords:** photonic crystal fiber, PMMA fiber, step-index fiber, power flow equation, bandwidth

## Abstract

By solving the time-dependent power flow equation, we present a novel approach for evaluating the bandwidth in a multimode step-index polymer photonic crystal fiber (SI PPCF) with a solid core. The bandwidth of such fiber is determined for various layouts of air holes and widths of Gaussian launch beam distribution. We found that the lower the NA of SI PPCF, the larger the bandwidth. The smaller launch beam leads to a higher bandwidth for short fibers. The influence of the width of the launch beam distribution on bandwidth lessens as the fiber length increases. The bandwidth tends to its launch independent value at a particular fiber length. This length denotes the onset of the steady state distribution (SSD). This information is useful for multimode SI PPCF applications in telecommunications and optical fiber sensing applications.

## 1. Introduction

**Figure 1:**
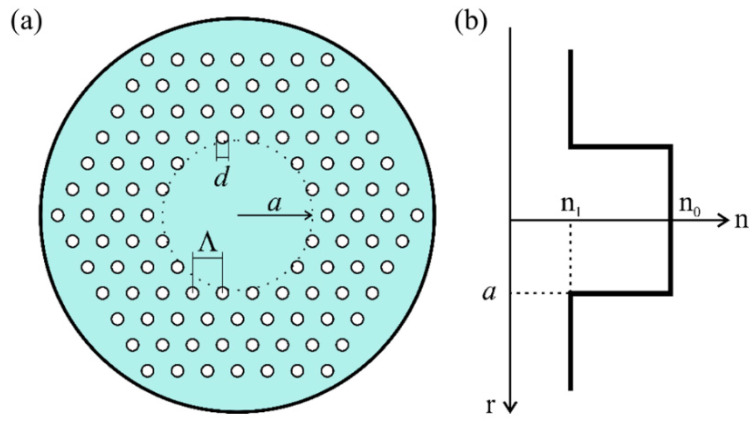
(**a**) Cross section of a multimode SI PPCF (Λ is the pitch, *d* is the diameter of air holes in the cladding); (**b**) RI profile of the proposed SI PPCF.

Selective stacking and chemical doping of materials have historically been employed for fabrication of optical fibers with different refractive-index (RI) distributions. Another method is to use a micro-structured pattern of very small holes that runs the length of the “holey” or PCFs. A PCF can have a solid core part and a holey cladding part, as shown in Figure 1. The hole pattern lowers the effective RI of the cladding, allowing the fiber to direct light. By selecting the hole pattern in the cladding throughout the design phase, the RI profile of the fiber can be modified. A variety of different micro-structured patterns of the PCF allows a broad versatility to modify its profile at the design stage [1,2,3,4,5,6,7]. A single-mode PCF has been produced for operation in a wide wavelength range [2]. The hollow core of a PCF, on the other hand, is also possible [8,9,10,11,12,13]. PCFs have been used in a variety of applications, including dispersion [14,15,16], supercontinuum production [17,18,19], birefringence [20], optofluidics [21], wavelength conversion [22,23] and sensing [24,25]. A typical numerical aperture of PCFs is NA = 0.5–0.6 [26,27,28,29,30]. With high NA PCFs, lensless beam focusing with the outstanding resolution has been recorded [31].

PCF propagation characteristics are influenced by differential mode attenuation, mode coupling, and modal dispersion. Light scattering in multimode optical fibers transfers power from one mode to another due to intrinsic perturbations, which causes mode coupling. Until recently, commercial simulation software packages were not designed for multimode PCFs. This deficiency is addressed in this paper for the first time, to our knowledge, by numerically solving the time-dependent power flow equation. The mode coupling properties of SI PPCF, and hence bandwidth, are affected by the parametric variance of the width of the launch beam distribution and the size of air holes. For three distinct widths of the launch beam distribution and sizes of air holes in the cladding, we estimated bandwidth in multimode SI PPCF (Poly(methyl methacrylate) or PMMA optical fibers) with solid core. The holes in the cladding are arranged in a triangular pattern with a uniform pitch (see Figure 1).

## 2. Design of SI PPCF

The SI PPCF is designed with air holes of uniform diameter in the cladding, which become a regular triangular lattice. The desirable effective RI is achieved by choosing the size (*d*) and pitch (Λ) of the cladding layer (Figure 1). The solid core region has the highest RI $n_{0}$. 

## 3. Time-dependent Power Flow Equation

The following time-dependent power flow equation [32] describes the time-dependent power flow for multimode optical fibers:

(1) $$\frac{\partial p(\theta ,z,t)}{\partial z}+\tau (\theta )\frac{\partial p(\theta ,z,t)}{\partial t}=-\alpha (\theta )P(\theta ,z,t)+\frac{1}{\theta }\frac{\partial }{\partial \theta }\left[D(\theta )\frac{\partial p(\theta ,z,t)}{\partial \theta }\right]$$

where *t* is time; p(θ,z,t) is the distribution of power over angle, space, and time; τ(θ) is mode delay per unit length; D(θ) is the mode-dependent coupling coefficient (usually assumed constant [32,33]); and $\alpha (\theta )=\alpha_{0}+\alpha_{d}(\theta )$ is the modal attenuation, where $\alpha_{0}$ represents conventional losses due to absorption and scattering. Except near cutoff, the attenuation is uniform $\alpha (\theta )=\alpha_{0}$ ($0\leq \theta \leq \theta_{m}$) [33] (it appears in the solution as the multiplication factor exp(–$\alpha_{0}$*z*) which also does not depend on *θ*). Therefore, α(θ) need not be accounted for when solving (1). In this paper for the first time, to our knowledge, by numerically solving the time-dependent power flow equation (1) we obtain bandwidth of the multimode SI PPCF.

## 4. Numerical Results and Discussion

For multimode solid-core SI PPCF, the bandwidth was examined for varying widths of launch beam distribution. For PCFs with air holes in a triangular lattice, the effective parameter *V* is given as:

(2) $$V=\frac{2\pi }{\lambda }a_{eff}\sqrt{n_{0}^{2}-n_{fsm}^{2}}$$

where $n_{0}$ is the RI of the core. The effective RI of the cladding part $n_{fsm}$ is the effective RI of fundamental space-filling mode in the triangular hole lattice, and $a_{eff}=\Lambda /\sqrt{3}$ [34]. The effective RI of the cladding $n_{1}=n_{fsm}$ can be obtained from equation (2), using the following equation [35]:

(3) $$V\left(\frac{\lambda }{\Lambda },\frac{d}{\Lambda }\right)=A_{1}+\frac{A_{2}}{1+A_{3}\exp\left(A_{4}\lambda /\Lambda \right)}$$

with the fitting parameters $A_{i}$ (*i* = 1 to 4):

(4) $$A_{i}=a_{i0}+a_{i1}{\left(\frac{d}{\Lambda }\right)}^{b_{i1}}+a_{i2}{\left(\frac{d}{\Lambda }\right)}^{b_{i2}}+a_{i3}{\left(\frac{d}{\Lambda }\right)}^{b_{i3}}$$

where the coefficients $a_{i0}$ to $a_{i3}$ and $b_{i1}$ to $b_{i3}$ (*i* = 1 to 4) are shown in Table 1.

**Figure 2:**
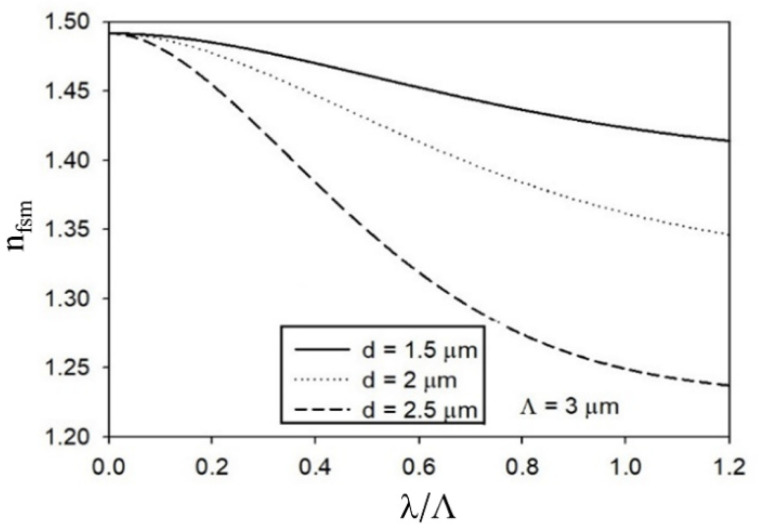
Effective RI of the inner cladding $n_{fsm}$ as a function of λ/Λ.

Figure 2 depicts the cladding’s effective RI $n_{1}\equiv n_{fsm}$ as a function of λ/Λ, for Λ=3 μm and for three values of *d*. Relevant values of the structural parameters of the analyzed multimode SI PPCF are summarized in Table 2, for λ = 645 nm.

**Figure 3:**
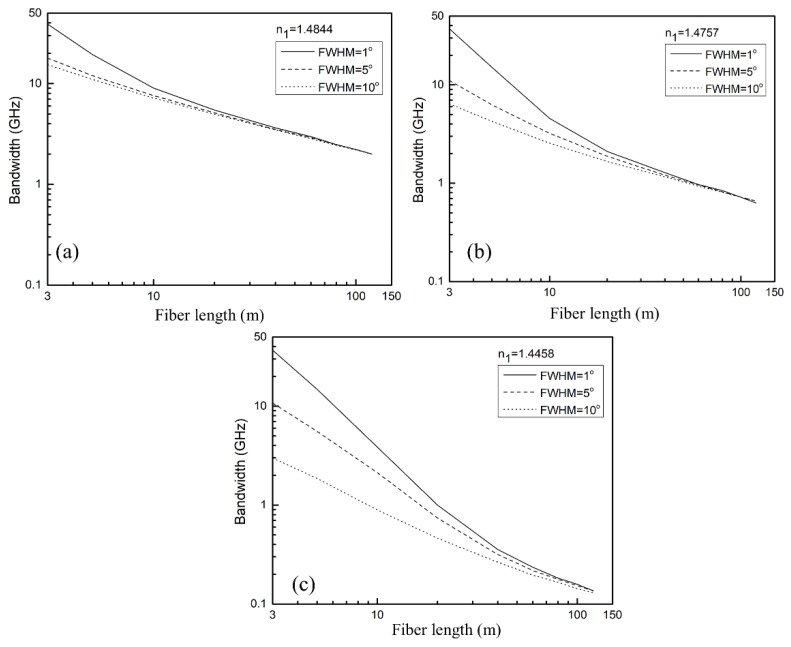
Bandwidth versus fiber length for multimode SI PPCF for three Gaussian launch beam widths with (FWHM)_z=0_ = 1°, 5°, and 10° for (**a**) *d* = 1 µm ($n_{1}$ = 1.4844), (**b**) *d* = 1.5 µm ($n_{1}$ = 1.4757) and (**c**) *d* = 2 µm ($n_{1}$ = 1.4458).

For the multimode SI PPCF with RI of the core $n_{0}=1.492$, core diameter 2a=0.980 mm, and optical fiber diameter b=1 mm, we solved the time-dependent power flow Equation (1) using a finite-difference method, assuming $D=1.649\times 10^{-4}\;{\mathrm{rad}}^{2}/m$ and $\alpha_{0}=0.22\;\mathrm{dB}/m$ [35,36]. We looked at impact of the diameters of air holes in the cladding of *d* = 1, 1.5, and 2 µm (i.e., the influence of NA of the fiber) and width of the launch beam distribution with (FWHM)_z=0_ = 1°, 5°, and 10° on the bandwidth. A detailed explanation of the numerical solution of the time-dependent power flow equation (1) is given in our previous work [37]. As illustration, Figure 3 shows the evolution of the bandwidth with fiber length calculated for three Gaussian launch beam distributions with (FWHM)_z=0_ = 1°, 5°, and 10° for the case with *d* = 1 µm ($n_{1}$ = 1.4844) (Figure 3a), *d* = 1.5 µm ($n_{1}$ = 1.4757) (Figure 3b) and *d* = 2 µm ($n_{1}$ = 1.4458) (Figure 3c). Figure 3 shows that the lower NA (larger $n_{1}$, smaller *d*), the higher bandwidth is obtained. In the case of the narrowest Gaussian launch beam, the highest bandwidth is observed at short optical fiber lengths. This is due to the guiding modes’ modal dispersion being reduced due to the narrower launch beam. The influence of the width of the launch beam distribution on bandwidth lessens as fiber length increases. Because mode coupling causes energy redistribution between guiding modes, the initial modal excitation (the FWHM of the launched beam) has a reduced impact on bandwidth for longer fibers. Figure 3 shows how bandwidth drops linearly for short lengths before switching to a 1/z^1/2^ functional dependence. This switch, and equilibrium mode distribution, occur at shorter optical fiber lengths for the wider Gaussian launch beam and lower NA. For (FWHM)_z=0_ = 1° this length is *L_c_* ≃ 5 m for $n_{1}$ = 1.4844, *L*_c_ ≃ 12.5 m for $n_{1}$ = 1.4757 and *L*_c_≃41 m for $n_{1}$ = 1.4458. For (FWHM)_z=0_ = 5° this length is *L_c_* ≃ 4.5 m for $n_{1}$ = 1.4844, *L*_c_ ≃ 11 m for $n_{1}$ = 1.4757 and *L*_c_ ≃ 39 m for $n_{1}$ = 1.4458. For (FWHM)_z=0_ = 10° this length is *L*_c_ ≃ 2.5 m for $n_{1}$ = 1.4844, *L*_c_ ≃ 9 m for $n_{1}$ = 1.4757 and *L*_c_ ≃ 33 m for $n_{1}$ = 1.4458 [36]. One can see that the shorter the length *L*_c_ results in the faster bandwidth improvement. The bandwidth tends to its launch independent value at a particular fiber length. This length denotes the onset of the SSD. It is worth noting that the proposed method for calculation of bandwidth in multimode SI PPCF can also be employed for multimode step-index silica PCFs.

## 5. Conclusions

By numerically solving the time-dependent power flow equation, we proposed a novel approach for evaluating the bandwidth in a multimode SI PPCF with a solid core and triangular air-hole lattice in the cladding. We showed that the lower the NA, the higher the bandwidth. The narrower Gaussian launch beam leads in increased bandwidth for short optical fibers. The influence of the width of the launch beam distribution on bandwidth lessens as fiber length increases. The bandwidth tends to its launch-independent value at a particular fiber length. This length denotes the onset of the steady state distribution. These customizable parameters allow for additional variety in the construction of multimode photonic crystal fibers. By changing the interplay between the material and geometrical dispersions, such design freedom in adjusting structural elements of the optical fiber for dispersion management.

## Figures and Tables

**Table 1:** Fitting coefficients in Equation (4).

	i=1	i=2	i=3	i=4
	0.54808	0.71041	0.16904	−1.52736
$a_{i1}$	5.00401	9.73491	1.85765	1.06745
$a_{i2}$	−10.43248	47.41496	18.96849	1.93229
$a_{i3}$	8.22992	−437.50962	−42.4318	3.89
$b_{i1}$	5	1.8	1.7	−0.84
$b_{i2}$	7	7.32	10	1.02
$b_{i3}$	9	22.8	14	13.4

**Table 2:** Effective RI of the cladding $n_{1}$, relative RI difference $\Delta =\left(n_{0}-n_{1}\right)/n_{0}$, where $n_{1}=1.492$, and the critical angle $\theta_{m}$ for varied *d* (air hole diameter) at 645 nm wavelength.

*d* (µm)	1.0	1.5	2.0
$n_{1}$	1.4844	1.4757	1.4458
$\Delta =\left(n_{0}-n_{1}\right)/n_{0}$	0.673673	0.677645	0.691611
$\theta_{m}$ (deg)	5.79	8.48	14.28

## Data Availability

The data presented in this study are available on request from corresponding author.
